# Ergot in the Treatment of Glactorrhœa and Mastitis

**Published:** 1876-06

**Authors:** 


Ergot in the Treatment of Glactorrhœa and Mastitis.—Dr. Schtscherbinenkoff observed, during an epidemic of raphania, that associated with the symptoms of ergotism, the secretion of milk in nursing women was often diminished or arrested. Recognizing the fact that an accumulation of milk in the ducts is the great cause of parenchymatous mastitis, he prescribed the ergot of rye for two women, who in former confinements had suffered from this trouble, giving it as soon as there was swelling of the glands. The results were most favorable. In so-called milk fever he also gave ergot, combined with quinine, in doses of grs. v.–x. of each three times a day. He also found it useful in arresting the secretion of milk after weaning the child. He gave as much as a drachm daily for a week, without unfavorable results.—*Sitzungsberichte d. Ges. russ, in Moskau*, 1875.

